# Update on the Basic Understanding of *Fusarium graminearum* Virulence Factors in Common Wheat Research

**DOI:** 10.3390/plants13081159

**Published:** 2024-04-22

**Authors:** Zeeshan Ali Buttar, Mengquan Cheng, Panqin Wei, Ziwei Zhang, Chunlei Lv, Chenjia Zhu, Nida Fatima Ali, Guozhang Kang, Daowen Wang, Kunpu Zhang

**Affiliations:** 1 State Key Laboratory of Wheat and Maize Crop Science, Center for Crop Genome Engineering, College of Agronomy, Henan Agricultural University, Zhengzhou 450002, China; 2 The Shennong Laboratory, Zhengzhou 450002, China; 3 Department of Plant Biotechnology, Atta-Ur-Rehman School of Applied Biosciences (ASAB), National University of Science and Technology, Islamabad 44000, Pakistan

**Keywords:** common wheat, grain yield, Fusarium head blight (Fhb), repeat-induced point mutation (RIP)

## Abstract

Wheat is one of the most important food crops, both in China and worldwide. Wheat production is facing extreme stresses posed by different diseases, including *Fusarium head blight* (FHB), which has recently become an increasingly serious concerns. FHB is one of the most significant and destructive diseases affecting wheat crops all over the world. Recent advancements in genomic tools provide a new avenue for the study of virulence factors in relation to the host plants. The current review focuses on recent progress in the study of different strains of *Fusarium* infection. The presence of genome-wide repeat-induced point (RIP) mutations causes genomic mutations, eventually leading to host plant susceptibility against *Fusarium* invasion. Furthermore, effector proteins disrupt the host plant resistance mechanism. In this study, we proposed systematic modification of the host genome using modern biological tools to facilitate plant resistance against foreign invasion. We also suggested a number of scientific strategies, such as gene cloning, developing more powerful functional markers, and using haplotype marker-assisted selection, to further improve FHB resistance and associated breeding methods.

## 1. Introduction

Bleached ears caused by the fungus *Fusarium graminearum* have emerged in luscious green fields of wheat, threatening wheat producers around the world [1]. After infection, the fungus spreads rapidly in the ear; continues to multiply and grow during the grain filling and maturity of wheat; and produces a variety of toxins in wheat grains, including deoxynivalenol (DON), nivalenol (NIV), and zearalenol (ZEN). Once these toxins enter the human body or livestock, they cause the body’s immunity to decline, leading to teratogenesis, cancer, abortion in pregnant women, and other pathogenic effects, thus posing serious harm to the health of humans and livestock. Historically, the first outbreak of this fungus was reported in 1884 in England, followed by America, Europe, Australia, and South Africa [2,3,4]. An estimated loss of USD 3 billion throughout the 1990s was documented in North America. Since then, approximately 28 million metric tons of wheat grain has been infected, accounting for almost USD 5.6 billion [1,5]. From 2004 to 2012, the annual economic losses ranged from CAD 1 million to 9 million in Alberta, Canada (Government of Alberta, 2015). 

Immature cereal grains become infected with the fungus at the anthesis stage. Thereafter, fungal hyphae spread within the spikelet through the vascular bundles of the rachis [6], thus promoting infection. In this case, one of the main factors influencing a plant’s susceptibility is thought to be the pathogen’s capacity to subvert its host’s physiological functions, including defense mechanisms, physiology, and basic metabolism, in order to make use of its resources. The two partners engage in intricate molecular cross-talk during these interactions, which includes the release of effectors, small secreted proteins with the ability to change the structure of host cells and target particular functions in host tissues which are also known as susceptibility factors. In response, the host plant activates a two-layered immune response [7]. The first layer is called pathogen-triggered immunity (PTI), which is activated when the plant recognizes the pathogen/microbial-associated molecular pattern (PAMP/MAMP) on the surface of the pathogen through the use of its pattern recognition receptors (PRRs) [8,9,10]. In order to curb this primary response, the pathogen release an arsenal of molecules, collectively called effector proteins. These molecules work as potential agents within the host to augment the capabilities through interfering with different cellular processes, such as signaling, cellular adherence, transcription, vesicular trafficking, membrane biogenesis, apoptosis, and metabolism [11]. However, some of these effectors are directly or indirectly detected by plants and deploy R-genes encoded with R-proteins, leading to rapid cell death; this is termed as the hypersensitive response (HR) or effector-triggered immunity (ETI) [11,12]. ETI is similar to the process of apoptosis in animals [13]. In contrast, resistance develops as depicted in Flor’s gene-for-gene model through the presence of similar R and effector proteins [14,15]. However, the proper delivery of these effector molecules is critical for the success of a pathogen. A small number of fungi, such as *Fusarium*, *Blumeria graminis*, *Puccina recondite*, *Puccinia graminis*, *Puccina striformis*, *Septoria nodorum*, and *Septoria tritici*, have been studied at this level. However, it is still difficult to uncover the multiple strategies of the mode of action of effectors, such as how the fungi maintain their effector protein evolutionary mechanism and what specific mechanism supports the pathogen in sustaining this evolutionary machinery in contrast with host plants. 

It has been suggested that repeat-induced point mutation (RIP) has an impact on genome duplication through gene duplication. Therefore, RIP is considered the most critical factor in the evolution of new functions [16,17]. In addition, epigenetic changes through genome-wide RIP mechanisms are a major weapon through which pathogens evolve in response to host plants. However, there is still a long way to go to understand the mechanism behind RIP. In this study, we specifically discuss and update the understanding of *Fusarium graminearum virulence* factors in common wheat, which may lead to overcoming the unknown strategies with which fungi maintain their evolutionary mechanism for completion with their host plant. 

## 2. Distinguished Features of *Fusarium graminearum* Life Cycle

**Figure 1:**
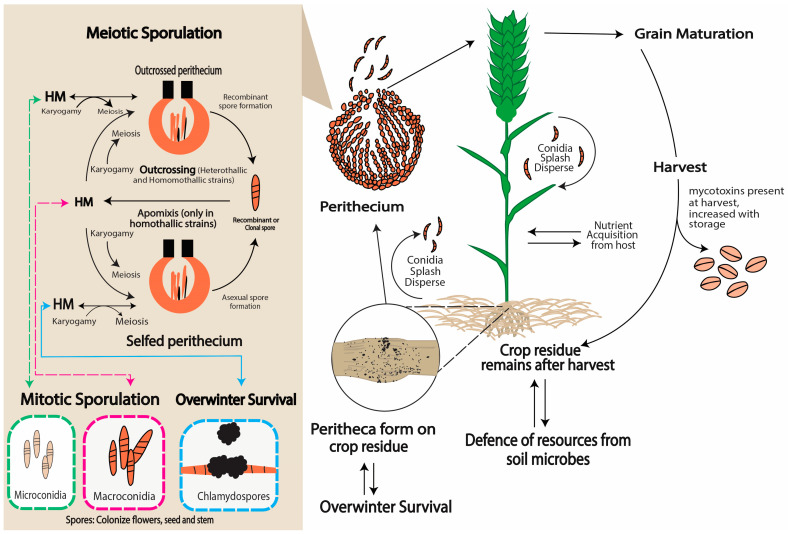
Generalized life cycle of *Fusarium* spp. [17]. Following plasmogamy and karyogamy, out-crossed and self-perithecium produce recombinant and clone meiotic spores, respectively. These form the haploid mycelium (HM), which, in turn, form three types of mitotic spores. While conidia (micro- or macro-conidia) can colonize the host, chlamydospores, in addition to direct colonization of the crop, can overwinter and develop into perithecium to restart the cycle when conditions are favorable.

Most of the *F. graminearum* life cycle is linked with the host plant (see Figure 1), and the fungus is haploid throughout much of its life cycle. The production of hyphae represents a bottleneck for sexual development within binucleate cells. Further, *F. graminearum* belongs to the phylum Ascomycota, which represents the binucleate phase known as the dikaryotic phase, in which two genetically separate nuclei remain coupled as new cells grow. It also possesses a homothallic nature, which means that it may produce sexual spores (ascospores) without the assistance of a sexually unique partner. This leads to the formation of binucleate cells (or “two nuclei cells”), which are genetically identical. Homothallism is caused by the haploid genome, including *Mat1-1* and *Mat1-2* genes from both mating types [17,18,19,20]. For example, *F. graminearum* colonizes the surfaces of wheat plants through the use of specialized unbranched hyphae known as runner hyphae (RH). Therefore, multi-celled complex appressoria known as infection cushions (IC) are developed, which accumulate an arsenal of (proven and putative) virulence factors to facilitate the invasion of epidermal cells [21,22].

## 3. *Fusarium graminearum* Germination Leading Up to Infection

The fungal plant pathogen initiates its first interaction with the host through spore germination. Spore germlings travel across the plant surface and utilize different invasion strategies to penetrate the host plant surfaces. These penetration strategies comprise pressurized melanized appressoria, which assists in physically punching through the plant cuticle, and non-melanized appressoria, which penetrates with the help of enzymes or cuticle damage to breach the plant surface. Further, the reactive oxygen species (ROS) level plays a significant role in the pathogen–host interaction. Thus, the *KatG2-mRFP* genes deliberately control the ROS level on the cell wall of invading hyphal cells. The pathogen delineates a change in infection by shifting the ROS levels through temporal and spatial regulation of *KatG2* to counteract the oxidative stress generated by host plant cells [20]. In contrast, over-expression of *DOHH* promotes ROS and sexual reproduction [23]. *FgMet3* and *FgMet14*, which are localized to the cytoplasm, have been identified in the synthesis of methionine and cysteine, and are further recognized to play a functional role in penetrability within the host plant [24]. Transcriptomic analysis of the *Tri* genes *Tri5*, *Tri6*, *Tri8*, *Tri9*, and *Tri14* indicated a significant lack of expression during conidial germination and plant penetration, whereas *Tri1*, *Tri3*, *Tri4*, *Tri7B*, *Tr10*, *Tri11*, and *Tri12* exhibited expression only at the first polar growth stage in wheat and barley. As such, it was deduced that the biosynthesis of DON by *F. graminearum* is not fully functional until after penetration [25]. The accumulation of DON in wheat seeds poses threats to human and animal health. In this case, linoleic acid (LA; cis-9, cis-12 C18:2) has been shown to have a significant role in wheat’s resistance to *F. graminearum* infection. It has been suggested that LA reinforces the cuticle, which acts as a barrier to pathogen entry [26]. Disruption of *FgLAI12* during wheat spike infection increases LA and SA levels to a higher extent in resistant wheat lines than in susceptible lines [27,28]. Therefore, *FgLAI12* is critical for mycelial growth and virulence in wheat. In addition, the *fg3_54* gene has been shown to facilitate the ability to infect wheat spikes through cell-to-cell penetration [29]. It has been deduced that effector proteins play significant roles in promoting pathogen manipulation in the host plant. 

## 4. Role of Effectors in Colonization of Host Plant

**Figure 2:**
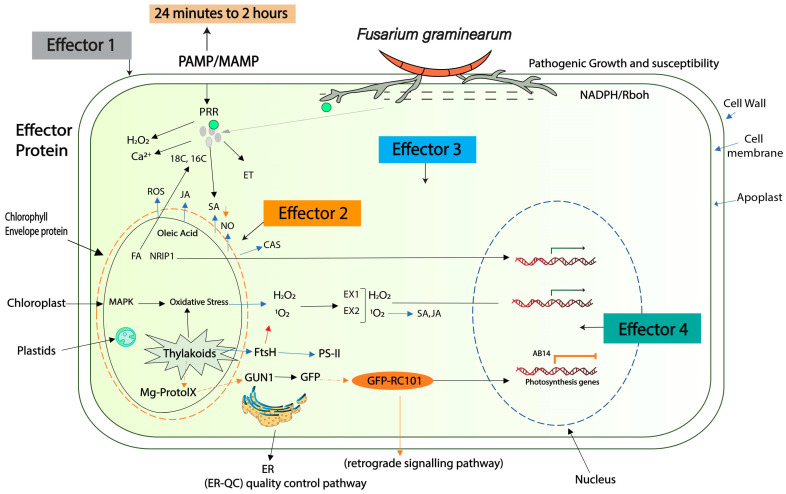
Schematic view of effectors-based mode of invasion at different cellular locations in relation to the host response. In PTI, various signaling events occur, including activation of influx of Ca^2+^ into the cytosol and production of ROS [40]. Effector 1 stimulates pathogen pressure at the host cell wall (e.g., leading to degradation), Effector 2 promotes cell-to-cell proliferation, and Effector 3 suppresses the cross-talk between PTI and ETI within the cytoplasm. Effector 4 leads to host genomic re-modeling, evidenced by different expression levels of *F. graminerum* effectors [36].

Effector proteins allow the pathogen to colonize the host plant through various mechanisms. These genes basically prevent recognition by the host, interfere with phytohormone defense pathways, regulate host gene expression, and influence host protein trafficking [30,31]; see Table S1. Effectors initially suppress the PAMPs response; for example, ROS production is the preliminary “PTI” signal against pathogen invasion, and a large number of ROS-related genes have been found to be activated in *F. graminearum* at 16 h after host invasion [32]. A detailed model of the ROS genes expressed at 16 hai [33] is shown in Figure 2. Similarly, peroxisomes are organelles that have been identified to play a role in ROS detoxification. *F. graminearum* secretes an effector, *Osp24*, which induces degradation of the wheat *TaSnRK1α* kinase to promote disease, while an orphan wheat protein, *TaFROG1*, can compete with *Osp24* for binding to *TaSnRK1α* and protect it from degradation [34]. The *PEX5* and *PEX6* genes from *F. graminearum* play a role in ROS detoxification, and deletion of *PEX5* and *PEX6* results in accumulation of ROS followed by necrotic cell death [35]. It has been suggested that peroxisomes are key organelles that balance ROS levels in filamentous fungi. Bioinformatics studies have identified 600 effectors secreted by *F. graminearum* [36], where 30 effector proteins showed interaction with a small cysteine-rich protein (SCPP) which contained N-terminal signal peptides and lacked transmembrane domains [36]. PRRs first recognize effectors to produce PTI, the failure of which results in a shift to the second layer of defense, ETI. *F. graminearum* has been identified to secrete plant-cell-wall-degrading enzymes composed of pectate lyases (cleave pectin), such as “*FG02386*, *FG03131*, *FG03483*, *FG03908*, and *FG04864*”, and xylanases (degrade xylan) such as “*FG00184*, *FG07639*, and *FG11304*”, which have significant contributions in the hemicellulose of monocot cell walls. These enzymes have been predicted to be involved as effector molecules that trigger host plant defense responses [37]. Therefore, they have important roles regarding acquisition and penetration of plant tissues [38]. *F. graminearum* has been shown to possess a higher number of plant necrosis-inducing proteins (or NIPs, also called NLPs), in contrast to *N. crassa* and *A. nidulans*. The first of the NLPs was a 24 KDa protein isolated from *F. oxysporum*, which has the capability to induce necrosis and ethylene biosynthesis in numerous dicotyledonous plants [39].

## 5. Host Plant Resistance Mechanism against Effector Invasion

Host plants have evolved a sophisticated two-layered defense system. PAMPs are recognized by PRRs, triggering the first PTI response, followed by a second layer comprising the cytoplasmic ETI response; failure of these responses results in ETS [41]. The plant susceptibility “S” factor has gained significant importance. The removal of “S” could lead toward resistance, as it indirectly supports the pathogen’s virulence mechanism [42,43,44,45]. Pathogens bombard a range of effector proteins into the host tissue through the formation of haustoria within the host plant, which are surrounded by a plant-derived extra-haustorial membrane (EHM) [42,46]. It has been suggested that the extra-haustorial membrane conducts effector screening before their release into the host plants. For example, in *F. graminearum*, 22 hexose transporters with various roles and specificities have been predicted, and have yet to be investigated [7,33,39,46]. In response, the host plant initiates NADPH-based ROS production, resulting in downstream signaling of PRRs and inducing Ca^2+^ influx [47]. Therefore, defense genes are named resistance (R) genes, which are deployed to protect against one or more pathogenic strains. R-genes most commonly encode NLRs, and resistance occurs when the NLR proteins identify an effector and subsequently stimulate efficient ETI. NLR genes have been observed, to some extent, in all resistance mechanisms. However, it is still a matter of debate whether all *NLR* genes in plant species can be genetically exposed as R genes. A typical NLR has a nucleotide-binding, *Apaf1*-resistant, CED4 (NB-ARC) central domain, as well as a leucine-rich repeat (LRR) C-terminal domain. Based on the N-terminal domain, NLRs can be further classified into three major classes: CC-type NLRs (RNLs), coiled-coil domain CC-NLRs (CNLs), and interleukin-1 receptor domain TIR-NLRs (TNLs) (112). In the case that NLRs bind with an effector or sense a modification of an effector target, a conformational change opens the P-loop of the NB-ARC domain to force the conversion of ADP to ATP. It has been speculated that this might be the mechanism used by NLR to trigger ETI [48]. The effectors that are recognized by R genes or NLRs are known as AVR genes or proteins. For example, *F. oxysporum*-secreted Avr2 was recognized by the tomato *1-2* (NB-LRR)-type resistance protein. However, point mutations prevent recognition [49]. Similarly, the introduction of a *1-3-mediated* resistance gene in tomato yielded resistance against *F. oxysporum (AVR1)* [50]. In addition, it is likely that *Osp24* suppression provides protection against FHB through interaction with *TaNACL-D1*. Two orphan proteins, *TaFROG* and *Osp24*, are found in hosts and pathogens, respectively. Therefore, during fungal–plant interactions, each might experience co-evolution. To the best of our knowledge, no other pathosystems have been reported regarding the active adoption of competing orphan proteins in either host plants or fungal pathogens. As they encode orphan proteins that are specifically expressed during infection, and expression-engineered *TaFROG* alleles with stronger interactions with *TaSnRK1α* or silencing *OSP24* may improve resistance against *F. graminearum* without yield penalties [34]. There remains significant pending work to determine the *F. graminearum* Avr-genes.

### 5.1. Effectors Inhibit R-Genes Expression through Chromatin Remodeling

Morphological changes are a very common and effective strategy used by pathogens to survive in the host plant. Therefore, during interaction with their host, pathogenic fungi undergo an array of morphological changes that are closely associated with numerous patterns in order to regulate their gene expression and biological processes [51]. In eukaryotic organisms, the genetic information comprises a specific nucleoprotein complex that is packed with an array of nucleosomes, known as chromatin. This is wrapped around a core of four histone proteins, namely, H2A, H2B, H3, and H4 [51,52]. In order to sense, environmental stimuli are integrated through epigenetic processes including chromatin remodeling, either allowing or inhibiting gene expression at the molecular level [52]. *F. graminearum* is a well-known hemi-biotroph fungus, and is the causative agent of FHB in cereal crops. For successful pathogen infection, each stage comprises specific genetic modifications [53], which are strictly associated with certain virulence mechanisms. These modifications are responsible for different post-translational modifications (PTMs) of histones, including acetylation, deacetylation, methylation, phosphorylation, ubiquitination, etc. Coordination between various histone modifiers defines a specific chromatin state of the genome [54]. Similarly, the immune response against pathogen infection requires extensive transcriptional reprogramming. Histone acetylation is considered to play a vital role in transcriptional regulation; for example, the cytoplasmic effector *PsAvh23* of the pathogen *Phytophthora sojae* has been identified as a modulator of histone acetyltransferase (HAT) in plants [55], resulting in host susceptibility through suppression of the immune response. Hence, histone modification (except in the case of methylation) could lead to alterations in the histone–histone or histone–DNA interactions. Furthermore, similar effects could be observed with respect to other histone proteins that could influence various changes in the function and structure of chromatin.

**Figure 3:**
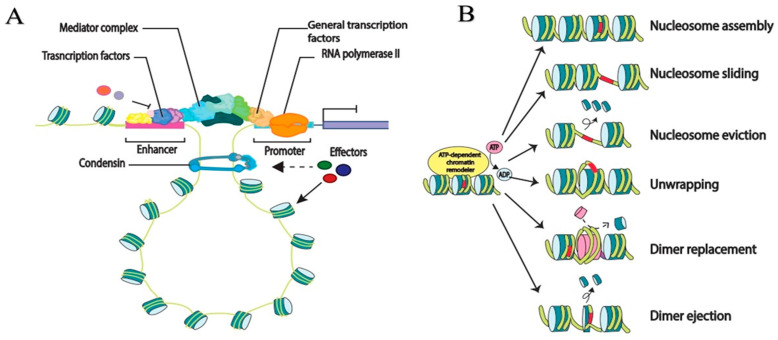
A schematic representation of the roles of effector proteins during fungal plant interaction. Histone modifications regulate chromatin compartmentalization via phase separation and influence gene expression. (**A**) DNA wraps around complexes of histone proteins, active enhancers regulate gene transcription through chromatin looping with the promoters of target genes, playing a role in the regulation of gene expression. (**B**) The nucleosome sliding expose a region that has been previously occluded; middle, ejection of a nucleosome to expose to corresponding DNA; and bottom, the substitution of a standard nucleosome with a variant histone.

Previous successful work has identified several signaling pathways and chromatin modifiers as being essential for fungal pathogenicity. Histone acetyltransferases (HATs), “addition of acetyl group”, and histone deacetylases (HDACs), “removal of acetyl group”, are the well-known PTMs that regulate the pattern of gene expression, and are schematically represented in Figure 3A,B. The balancing act between these responses is important for appropriate cellular development and function [55]. HATs can be sub-divided into five sub-families based on conserved motifs: *MYST* (*MOZ*, *Tip60*, *Sas2*, *Ybf2/Sas3*), *GNAT* (Gcn5-related N-acetyltransferases), *p300*/*CBP*, basal transcription factor (including TFIID), and nuclear receptor cofactors. They can be further divided based on their cellular location in nuclear type-A or cytoplasmic type-B HATs, regardless of which family they belong to. A summary of their sub-cellular locations with Go-annotation is provided in Table 1.

In contrast, HDACs reverse the activity of HATs through removing acetylation on the ε-amino group of lysine residues. Thus, HDACs have been observed to be transcriptional repressors. They restore the positive charge of lysine, rendering the underlying DNA sequences relatively inaccessible to transcriptional machinery through stabilization of the local chromatin structure, as explained in Figure 3A. HDACs have been classified into four sub-classes based on phylogenetic analysis and sequence homology [56]. In previous work, successful degradation of *F. graminearum* histone acetylation at *H2BK11*, *H3K11*, *H3K14*, *H3K18*, and *H3K27* has significantly reduced fungal growth, virulence, and mycotoxin biosynthesis [51,57]. Considering the results of previous studies, the transcription factor *FgPacC* plays a significant role in protecting the fungus from iron toxicity through direct binding of promoters and inhibition of SAGA activity [58]; however, there is still a need to carry out significant research to observe the accurate mechanisms through which the morphological changes and virulence factors of the pathogen are controlled. Furthermore, several defense factors, including phenolics, alkaloids, polyacetylenes, hydrogen peroxide (H_2_O_2_), and a series of pathogenesis-related (PR) compounds, may be produced by the plant. Therefore, epigenetic modification within the host genome through pathogen invasion may significantly suppress the production of plant defense proteins. For example, in the case of *Blumeria graminis f.* sp *tritici* (*Bgt*) causing powdery mildew in bread wheat (*Triticum aestivum* L.), significant improvements in *TaPR1*, *TaPR2*, *TaPR5*, and *TaWRKY45* expression were observed through silencing of *TaHBT701*, *TaHDA6*, and *TaHOS15* [59]. It can be speculated that their resistance mechanism might be suppressed by *F. graminearum*. It has been observed that *MsDef1* and *MtDef4* are produced in *Medicago* spp. to resist against *F. graminearum* growth [60].

### 5.2. Effector Protein Evolution through Repeat-Induced Point Mutation 

**Figure 4:**
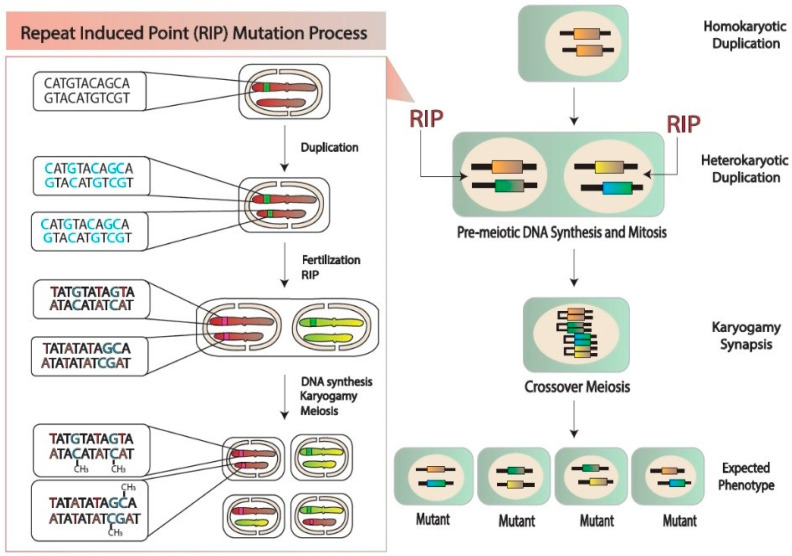
Potential patterns of mutations by repeat-induced point (RIP) immediately before karyogamy of *F. graminearum*. This fungus differs from other filamentous fungi as it is homothallic (self-fertile) and rarely out-crosses, which limits the opportunity to acquire new repeats [18,78]. RIP identifies duplicated sequences and introduces C: G to T: A transition mutations.

Transposable elements (TEs) are considered a major factor in genome expansion, and are spread through the genome through a self-copying mechanism [61,62]. At the same time, TEs pose a severe threat to the pathogen’s genome, as the effector genes are commonly located in TE-rich regions, with epigenetic regulations observed in an extensive range of crop pathogens [62]. Therefore, the expression of effector genes is mostly controlled through variation in the heterochromatin state upon host–pathogen infection. In this scenario, to mitigate the adverse effects of “self-repeat” sequencing, numerous filamentous fungi species use a genome-wide defense system known as the repeat-induced point (RIP) mutation [63]. Further, the point mutation stops the translation of DNA such as the amino acid glycine, which may be changed to a stop codon, causing the proteins to be unable to complete the intended tasks. The problem arises during the processes of transcription and replication of DNA. These changes stop the cell from reproducing and thus lead to the death of the cell. For example, the *Leptoshaeria maculans* genome comprises one-third AT and contains effector genes and TEs families, both of which are affected by RIP. This novel mechanism for effector genes promotes rapid sequence diversification and allows the fungus to adapt rapidly to novel host-derived constraints [64]. Therefore, it is considered to be one of the main fungal-specific genome defense mechanisms. Point mutation of G: C to A: T at a very high rate is introduced, leading to non-functional TE copies [19,65]. By consequently reducing the GC content in the affected sequence, further RIP promotes a large block of evolution through gene duplication [66]. However, it restricts pathogen effector evolution, which is mostly prone to duplication due to the repetitive nature of their chromosomal locations [67]. Selker et al. conducted a pioneering study in *Neurospora crassa* to identify RIP [68,69]. Some key characteristics of RIP have been defined in *N. crassa* [70,71], as demonstrated in Figure 4. Hokyoung et al. confirmed RIP in *Gibberella zea* (anamorph: *F. graminearum*) [72]. *F. graminearum* has a homothallic (self-fertile) nature, which differentiates it from other filamentous fungi, and it rarely out-crosses to other strains, thus limiting it to gaining new repeats [73]. In addition, the presence of a genome-wide RIP mechanism limits *F. graminearum* to acquiring repetitive sequences. RIP permanently mutates the cytosine in duplicated motifs, inducing adenine- and thymine-biased transition mutation in the target sequences [74]. Likewise, transposon mutation during the sexual cycle of *F. graminearum* introduces C: G to T: A [75,76]. Further, RIP has been explored through epigenetic silencing in *N. crassa*. The entire process occurs during sexual reproduction, prior to karyogamy and meiosis, when two copies of the genome are still present in the dikaryotic cell. Therefore, RIP is thought to be the most clear-cut example of a genome defense mechanism, as it has no other known purpose [77]. The genomes of a number of species or subspecies of more recent and ancient outgroups that shared a common ancestor will be sequenced in order to test the validity of the proposed evolution scenario of RIP and provide additional information on the origin of the effectors, genome invasion by TEs, and the subsequent effect on effector generation and diversification. 

## 6. Important Unresolved Questions

The host’s innate immune system mostly recognizes cysteine-rich proteins. In the case of *F. graminearum*, 76 peptides with less than 200 aa contain at least 4% cysteine residues, which may be speculated as the possible reason for PTI failure against *F. graminearum*. As previously identified, avirulence effectors of fungi are small, cysteine-rich proteins [79,80]. Therefore, further investigation is required in order to address related questions. In previous work, wheat histone (H1-H4) has been found to be involved in the *F. graminearum* resistance mechanism [59,81,82], which raises a question related to the histone–histone protein interactions taking place between *F. graminearum* and wheat histone protein, as well as how this may stimulate wheat susceptibility, which remains a very interesting and elusive question. 

### 6.1. Molecular Pathogenicity

Necrotrophic pathogens feed and live on dying tissue, in contrast to biotrophic pathogens. Their resistance mechanism has been investigated in association with the wall-associated kinase class of receptors, which drives the resistance pathway against the biotrophic pathogen. Necrotrophic pathogens such as *Phaeosphaeria nodorum* have been observed to hijack the host molecular pathways involved in the resistance mechanism, demonstrating the complex nature of susceptibility and resistance in the interactions between necrotrophic or biotrophic pathogens and plants [83]. Therefore, to gain access to the host cell, pathogens either use natural opening (e.g., stomata), wounds, the aid of vectors, or utilize an arsenal of cell-wall-degrading enzymes (CWDEs) to deconstruct the structural components of host CW, assisting penetration and diffusion in the host tissues, and at the same time providing carbon sources and promoting leakage of nutrients from the protosplast [83]. Therefore, we can speculate the possible involvement of *F. graminearum* in hijacking the host wall-associated kinase class through the release of cell-wall-degrading enzymes [84,85,86], resulting in a lack of immune response against *F. graminearum* infection. Further, co-variation of wheat and fungal protein accumulation at different time points indicated that *F. graminearum* effector proteins intercept the molecular mechanisms, thus determining the fate of the interaction [85]. WRKY transcription factors are members of the large protein family. In a previous study, *TaWRKY45* was found to be up-regulated in response to benzothiadiazole (BTH), a plant immune system strengthener [86]. Here, we propose that histone modification from HAT to HDAC suppressed *TaWRKY45* expression, resulting in a lack of immunity response against FHB. The histone–histone interaction, as seen in Figure 3B, caused modifications in wheat histone (H1-H4) proteins, resulting in susceptibility of the wheat. Furthermore, histone proteins have been linked with a resistance mechanism against *F. graminearum* [86].

### 6.2. How Can We Develop Resistant Cultivars More Efficiently?

**Figure 5:**
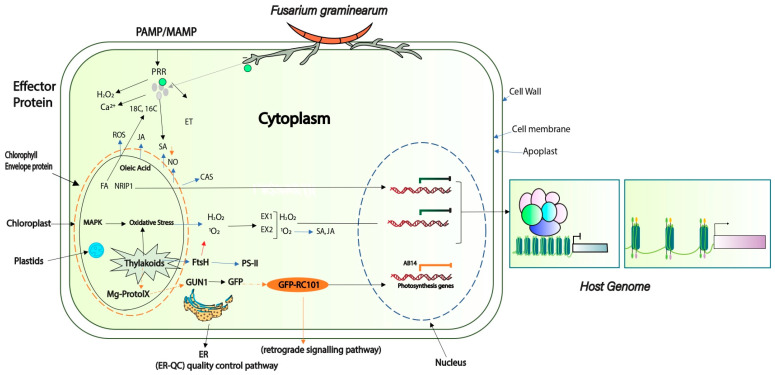
Proposed model of plant resistance. Epigenetic changes within the host genome result in silencing of resistance genes against foreign invasion, which is why it is important to protect the host genome against effector interaction. This will protect the host genome, allowing it to promote its resistance mechanism against the pathogen.

Previous studies have speculated on the negative correlation between FHB severity, plant height, and anther exclusion. It has been suggested that tall genotypes without anthers could have some level of resistance against FHB [87,88,89,90,91]. Recent studies have uncovered the underlying mechanism of glutathione S-transferase (GST) encoded by Fhb7. The *Fhb7* is conserved in Epichloë species and can detoxify the trichothecene mycotoxins secreted by Fusarium species. Thus, integration of *Fhb7* into a plant genome could be beneficial in eliminating the need for symbiotic association per se. The finding of Fhb7-mediated resistance to both FHB and crown rot diseases further emphasizes the importance of the use of *Thinopyrum elongatum*, a species used in distant hybridization breeding of wheat. Therefore, further thorough investigation is required when using genetic material in resistance breeding, as shown in Figure 5.

### 6.3. Climate Change on FHB Disease and Solution

In addition to the susceptible host, multiple environmental factors, including location, temperature, humidity, salinity, flooding, and drought, play vital roles in regulating the pathogenicity of FHB. In addition to this, the varying atmospheric CO_2_ levels also affect the spread of FHB. The *F. graminearum* infection is promoted by environmental changes that are related to the induction of DON production. A high level of DON production related to sucrose, 1-kestose, and nystose was observed in *F. graminearum*. Further, the carbon catabolic repression negatively regulates the expression of genes required for utilizing carbon sources. Therefore, predicted binding sites of *FgCreA* are present in promoters of *TRI1*, *TRI3-TRI8*, *TRI10*, *TRI12*, and *TRI101* [81,86]. However, the extent of the effect of *FgCreA* on the regulation of DON biosynthesis is still unknown in relation to carbon and other environmental factors. 

### 6.4. Strengthen the Study of Molecular Mechanisms of Pathogenic Bacteria’s Pathogenesis

Strong evidence of susceptible sites and genes may further strengthen the investigation of the molecular mechanism underlying the pathogenesis, growth, and development of pathogenic bacteria. Therefore, effective use can be made of gene editing technology, such as gene silencing induced in the host. Host-induced gene silencing (HIGS) technology has been applied for wheat scab resistance breeding, such as the scab resistance gene *TaHRC* [92], where the wild type is a pathogen-sensitive gene which produces resistance to FHB after mutation. This kind of gene can be mutated accurately in FHB-sensitive varieties through the use of gene editing technology, changing the susceptible genes into disease-resistant genes.

## Figures and Tables

**Table 1:** *Fusarium graminearum* name or ID of histone modification factors.

Protein Name or ID	Gene Family	Sub-Cellular Location	Go Term Annotation
*FGSG_02040T0*	HAT_GNAT >> ELP3	Nuclear, cytoplasmic	** GO:0008080**: N-acetyltransferase activity, ** GO:0046933**: hydrogen-transporting ATP synthase activity, rotational mechanism
*FGSG_00280T0*	HAT_GNAT >> GCN5	nuclear	** GO:0008080**: N-acetyltransferase activity
*FGSG_04254T0*	HAT_MYST >> Esa1	nuclear	Not found
*FGSG_06047T0*	HAT_MYST >> Sas2	nuclear	Not found
*FGSG_08481T0*	HAT_MYST >> Tip60	nuclear	** GO:0005515**: protein binding, ** GO:0007242**: intracellular signaling cascade ** GO:0006355**: regulation of transcription, DNA-dependent ** GO:0008270**: zinc ion binding
*FGSG_02567T0*	HMT >> DOT1	nuclear	Not found
*FGSG_01134T0*	HMT >> PRMT_1	Mitochondrial, cytoplasmic	** GO:0008757**: S-adenosylmethionine-dependent methyltransferase activity ** GO:0005737**: cytoplasm ** GO:0006479**: protein amino acid methylation ** GO:0008276**: protein methyltransferase activity
*FGSG_10718T0*	HMT >> PRMT_1	cytoplasmic	** GO:0008757**: S-adenosylmethionine-dependent methyltransferase activity
*FGSG_07445T0*	HMT >> SET1	nuclear	** GO:0000166**: nucleotide binding
*FGSG_05558T0*	HMT >> SET2	nuclear	Not found
*FGSG_01558T0*	HDM >> JHDM3_JMJD2	Nuclear	** GO:0005515**: protein binding ** GO:0006355**: regulation of transcription, DNA-dependent ** GO:0008270**: zinc ion binding
*FGSG_00780T0*	HDAC >> ClassI	Nuclear	** GO:0016575**: histone deacetylation ** GO:0004407**: histone deacetylase activity ** GO:0005634**: nucleus
*FGSG_01353T0*	HDAC >> ClassI	Cytoplasmic, nuclear	** GO:0016575**: histone deacetylation ** GO:0004407**: histone deacetylase activity ** GO:0005634**: nucleus
*FGSG_04324T0*	HDAC >> ClassIIB	Cytoplasmic, nuclear	Not found
*FGSG_09218T0*	HDAC >> ClassIII	Nuclear, cytoplasmic	** GO:0006342**: chromatin silencing ** GO:0003677**: DNA binding ** GO:0006355**: regulation of transcription, DNA-dependent ** GO:0005677**: chromatin silencing complex

## Supplementary Materials

The following supporting information can be downloaded at: https://www.mdpi.com/article/10.3390/plants13081159/s1, Table S1: Previously studied effector genes of Fusarium graminearum, including their sub-cellular location, gene function, and phenotype mutation.

## References

[1] Hao Y., Rasheed A., Zhu Z., Wulff B.B.H., He Z. (2020). Harnessing Wheat *Fhb1* for *Fusarium* Resistance. Trends Plant Sci..

[2] Stack R.W., Leonard K.J., Bushnell W.R. (2003). History of *Fusarium* Head Blight with Emphasis on North America. Fusarium Head Blight of Wheat and Barley.

[3] McMullen M., Leonard K.J., Bushnell W.R. (2003). Impacts of *Fusarium* Head Blight on the North American Agriculture Community: The Power of One Disease to Catapult Change. Fusarium Head Blight of Wheat and Barley.

[4] Burgess L.W., Klein T.A., Bryden W.L., Tobin N.F. (1983). Head Blight of Wheat Caused by *Fusarium-Graminearum* group 1 in New South Wales Australia. Australas. Plant Pathol..

[5] Wulff B.B.H., Jones J.D.G. (2020). Breeding a Fungal Gene into wheat. Science.

[6] Parry D.W., Jenkinson P., McLeod L. (1995). *Fusarium* Ear Blight (scab) in Small Grain Cereals-a Review. Plant Pathol..

[7] Jones J.D.G., Dangl J.L. (2006). The plant immune system. Nature.

[8] Schulze S., Yu L., Hua C., Zhang L., Kolb D., Weber H., Ehinger A., Svenja C., Stahl M., Franz-Wachtel M. (2022). The Arabidopsis TIR-NBS-LRR Protein CSA1 Guards BAK1-BIR3 Homeostasis and Mediates Convergence of Pattern- and Effector-induced Immune responses. Cell Host Microbe.

[9] Zipfel C. (2014). Plant Pattern-recognition Receptors. Trends Immunol..

[10] Balint-Kurti P. (2019). The Plant Hypersensitive Response: Concepts, Control and Consequences. Mol. Plant Pathol..

[11] Blümke A., Falter C., Herrfurth C., Sode B., Bode R., Schäfer W., Feussner I., Voigt C.A. (2014). Secreted fungal effector lipase releases free fatty acids to inhibit innate immunity-related callose formation during wheat head infection. Plant Physiol..

[12] Yang C.C., Wang Z.Y., Cheng C.M. (2023). Insights into Superinfection Immunity Regulation of Xanthomonas Axonopodis *Filamentous Bacteriophage* cf. Curr. Microbiol..

[13] Petit-Houdenot Y., Fudal I. (2017). Complex Interactions between Fungal Avirulence Genes and Their Corresponding Plant Resistance Genes and Consequences for Disease Resistance Management. Front. Plant Sci..

[14] Ausubel F.M. (2005). Are Innate Immune Signaling Pathways in Plants and Animals Conserved?. Nat. Immunol..

[15] Flor H.H. (1971). Current Status of the Gene-for-gene Concept. Annu. Rev. Phytopathol..

[16] Flor H.H. (1942). Inheritance of Pathogenicity in *Melampsora lini*. Phytopathology.

[17] Trail F. (2009). For Blighted Waves of Grain: *Fusarium graminearum* in the Postgenomics Era. Plant Physiol..

[18] Van W.S., Wingfield B.D., de Vos L., van der Merwe N.A., Santana Q.C., Steenkamp E.T. (2019). Repeat-Induced Point Mutations Drive Divergence between *Fusarium* Circinatum and its Close Relatives. Pathogens.

[19] Klix V., Nowrousian M., Ringelberg C., Loros J.J., Dunlap J.C., Pöggeler S. (2010). Functional Characterization of MAT1-1-specific Mating-type Genes in the Homothallic Ascomycete *Sordaria macrospora* Provides New Insights into Essential and Nonessential Sexual Regulators. Eukaryot Cell.

[20] Guo Y., Yao S., Yuan T., Wang Y., Zhang D., Tang W. (2019). The Spatiotemporal Control of KatG2 Catalase-peroxidase Contributes to the Invasiveness of *Fusarium graminearum* in Host Plants. Mol. Plant Pathol..

[21] Yun S.H., Arie T., Kaneko S., Yoder O.C., Turgeon B.G. (2000). Molecular Organization of Mating Type Loci in Heterothallic, Homothallic and Asexual *Gibberella*/*Fusarium* species. Fungal Genet. Biol..

[22] Mentges M., Glasenapp A., Boenisch M., Malz S., Henrissat B., Frandsen R.J.N., Güldener U., Münsterkötter M., Bormann J., Lebrun M.H. (2020). Infection Cushions of *Fusarium graminearum* are Fungal Arsenals for Wheat Infection. Mol. Plant Pathol..

[23] Ofek P., Yeini E., Arad G., Danilevsky A., Pozzi S., Luna C.B., Dangoor S.I., Grossman R., Ram Z., Shomron N. (2023). Deoxyhypusine hydroxylase: A Novel Therapeutic Target Differentially Expressed in Short-term vs Long-term Survivors of Glioblastoma. Int. J. Cancer.

[24] Zhao F., Yuan Z., Wen W., Huang Z., Mao X., Zhou M., Hou Y. (2022). FgMet3 and FgMet14 Related to Cysteine and Methionine Biosynthesis Regulate Vegetative Growth, Sexual Reproduction, Rathogenicity, and Sensitivity to Fungicides in *Fusarium graminearum*. Front. Plant Sci..

[25] Villafana R.T., Ramdass A.C., Rampersad S.N. (2019). Selection of *Fusarium* Trichothecene Toxin Genes for Molecular Detection Depends on TRI Gene Cluster Organization and Gene Function. Toxins.

[26] Zhang Y.Z., Wei Z.Z., Liu C.H., Chen Q., Xu B.J., Guo Z.R., Cao Y.L., Wang Y., Han Y.N., Chen C. (2017). Linoleic Acid Isomerase Gene FgLAI12 Affects Sensitivity to Salicylic Acid, Mycelial Growth and Virulence of *Fusarium graminearum*. Sci. Rep..

[27] Martinez-Rocha A.L., Woriedh M., Chemnitz J., Willingmann P., Kröger C., Hadeler B., Hauber J., Schäfer W. (2016). Posttranslational Hypusination of the Eukaryotic Translation Initiation Factor-5A Regulates *Fusarium graminearum* Virulence. Sci. Rep..

[28] Ellinger D., Sode B., Falter C., Voigt C.A. (2014). Resistance of Callose Synthase Activity to Free Fatty acid Inhibition as an Indicator of *Fusarium* Head Blight Resistance in Wheat. Plant Signal Behav..

[29] Jia L.J., Tang H.Y., Wang W.Q., Yuan T.L., Wei W.Q., Pang B., Gong X.M., Wang S.F., Li Y.J., Zhang D. (2019). A Linear Nonribosomal Octapeptide from *Fusarium graminearum* Facilitates Cell-to-cell Invasion of Wheat. Nat. Commun..

[30] Kamoun S.A. (2006). Catalogue of the Effector Secretome of Plant Pathogenic Oomycetes. Annu. Rev. Phytopathol..

[31] Kamoun S. (2007). Groovy times: Filamentous Pathogen Effectors Revealed. Curr. Opin. Plant Biol..

[32] Zhang X.W., Jia L.J., Zhang Y., Jiang G., Li X., Zhang D., Tang W.H. (2012). In Planta Stage-specific Fungal Gene Profiling Elucidates the Molecular Strategies of *Fusarium graminearum* Growing inside Wheat Coleoptiles. Plant Cell.

[33] Kugler K.G., Siegwart G., Nussbaumer T., Ametz C., Spannagl M., Steiner B., Lemmens M., Mayer K.F., Buerstmayr H., Schweiger W. (2013). Quantitative Trait Loci-dependent Analysis of a Gene Co-expression Network Associated with *Fusarium* Head Blight Resistance in Bread Wheat (*Triticum aestivum* L.). BMC Genom..

[34] Jiang C., Hei R., Yang Y., Zhang S., Wang Q., Wang W., Zhang Q., Yan M., Zhu G., Huang P. (2020). An Orphan Protein of *Fusarium graminearum* Modulates Host Immunity by Mediating Proteasomal Degradation of *TaSnRK1α*. Nat. Commun..

[35] Min K., Son H., Lee J., Choi G.J., Kim J.C., Lee Y.W. (2012). Peroxisome Function Is Required for Virulence and Survival of *Fusarium graminearum*. Mol. Plant Microbe Interact..

[36] Hao G., McCormick S., Usgaard T., Tiley H., Vaughan M.M. (2020). Characterization of Three *Fusarium graminearum* Effectors and Their Roles During *Fusarium Head Blight*. Front. Plant Sci..

[37] Walton J.D. (1994). Deconstructing the Cell Wall. Plant Physiol..

[38] Wang Q., Han C., Ferreira A.O., Yu X., Ye W., Tripathy S., Kale S.D., Gu B., Sheng Y., Sui Y. (2011). Transcriptional Programming and Functional Interactions within the *Phytophthora sojae* RXLR Effector Repertoire. Plant Cell.

[39] Brown N.A., Antoniw J., Hammond-Kosack K.E. (2012). The Predicted Secretome of the Plant Pathogenic Fungus *Fusarium graminearum*: A Refined Comparative Analysis. PLoS ONE.

[40] Köster P., DeFalco T.A., Zipfel C. (2022). Ca^2+^ Signals in Plant Immunity. EMBO J..

[41] Gebrie A. (2023). Transposable Elements as Essential Elements in the Control of Gene Expression. Mob. DNA.

[42] Lu S., Edwards M.C. (2016). Genome-wide Analysis of Small Secreted Cysteine-rich Proteins Identifies Candidate Effector Proteins Potentially Involved in *Fusarium graminearum*-wheat interactions. Phytopathology.

[43] Lapin G., Van den Ackerveken G. (2013). Susceptibility to Plant Disease: More than a Failure of Host Immunity. Trends Plant Sci..

[44] Van Schie C.C., Takken F.L. (2014). Susceptibility Genes: How to be a Good Host. Annu. Rev. Phytopathol..

[45] Zaidi S.S., Mukhtar M.S., Mansoor S. (2018). Genome Editing: Targeting Susceptibility Genes for Plant Disease Resistance. Trends Biotechnol..

[46] Guenther J.C., Hallen-Adams H.E., Bücking H., Shachar-Hill Y., Trail F. (2009). Triacylglyceride Metabolism by *Fusarium graminearum* During Colonization and Sexual Development on Wheat. Mole. Plant Microbe Interact..

[47] Hao G., Tiley H., McCormick S. (2022). Chitin Triggers Tissue-Specific Immunity in Wheat Associated with *Fusarium Head Blight*. Front. Plant Sci..

[48] Zuo N., Bai W.Z., Wei W.Q., Yuan T.L., Zhang D., Wang Y.Z., Tang W.H. (2022). Fungal CFEM Effectors Negatively Regulate a Maize Wall-associated Kinase by Interacting with its Alternatively Spliced Variant to Dampen Resistance. Cell Rep..

[49] Houterman P.M., Ma L., van Ooijen G., de Vroomen M.J., Cornelissen B.J.C., Takken F.L.W., Rep M. (2009). The Effector Protein *Avr2* of the Xylem-colonizing Fungus *Fusarium Oxysporum* Activates the Tomato Resistance Protein I-2 Intracellularly. Plant J..

[50] Catanzariti A.M., Lim G.T.T., Jones D.A. (2015). The Tomato I-3 Gene: A Novel Gene for Resistance to *Fusarium* wilt Disease. New Phytol..

[51] Kong X., van Diepeningen A.D., van der Lee T.A.J., Waalwijk C., Xu J., Xu J., Zhang H., Chen W., Feng J. (2018). The *Fusarium graminearum* Histone Acetyltransferases Are Important for Morphogenesis, DON Biosynthesis, and Pathogenicity. Front. Microbiol..

[52] Luger K., Rechsteiner T.J., Flaus A.J., Waye M.M.Y., Richmond T.J. (1997). Characterization of Nucleosome Core Particles Containing Histone Proteins Made in Bacteria. J. Mol. Biol..

[53] Badeaux A.I., Shi Y. (2013). Emerging Roles for Chromatin as a Signal Integration and Storage Platform. Nat. Rev. Mol. Cell Biol..

[54] Lanver D., Muller A.N., Happel P., Schweizer G., Haas F.B., Franitza M., Pellegrin C., Reissmann S., Altmuller J., Rensing S.A. (2018). The Biotrophic Development of Ustilago Maydis Studied by RNA-Seq Analysis. Plant Cell.

[55] Kong L., Qiu X., Kang J., Wang Y., Chen H., Huang J., Qiu M., Zhao Y., Kong G., Ma Z. (2017). A *Phytophthora* Effector Manipulates Host Histone Acetylation and Reprograms Defense Gene Expression to Promote Infection. Curr. Biol..

[56] Ori N., Eshed Y., Paran I., Presting G., Aviv D., Tanksley S., Zamir D., Fluhr R. (1997). The I2C Family from the Wilt Disease Resistance Locus I2 Belongs to the Nucleotide Binding, Leucine-rich Repeat Superfamily of Plant Resistance Genes. Plant Cell.

[57] Rep M., Meijer M., Houterman P.M., van der Does H.C., Cornelissen B.J.C. (2005). *Fusarium oxysporum* Evades I-3-Mediated Resistance without Altering the Matching Avirulence Gene. Mol. Plant Microbe Interact..

[58] Gu Q., Wang Y., Zhao X., Yuan B., Zhang M., Tan Z., Zhang X., Chen Y., Wu H., Luo Y. (2022). Inhibition of Histone Acetyltransferase GCN5 by a Transcription Factor FgPacC Controls Fungal Adaptation to Host-derived Iron Stress. Nucleic Acid Res..

[59] Zhi P., Kong L., Liu J., Zhang X., Wang X., Li H., Sun M., Li Y., Chang C. (2020). Histone Deacetylase TaHDT701 Functions in TaHDA6-TaHOS15 Complex to Regulate Wheat Defense Responses to *Blumeria graminis* f. sp.. tritici. Int. J. Mol. Sci..

[60] Sagaram U.S., Pandurangi R., Kaur J., Smith T.J., Shah D.M. (2011). Structure-activity Determinants in Antifungal Plant Defensins MsDef1 and MtDef4 with Different Modes of Action against *Fusarium graminearum*. PLoS ONE.

[61] Fouché S., Oggenfuss U., Chanclud E., Croll D.A. (2022). Devil’s Bargain with Transposable Elements in Plant Pathogens. Trends Genet..

[62] Daboussi M.J., Capy P. (2003). Transposable Elements in Filamentous Fungi. Annu. Rev. Microbiol..

[63] Van Wyk S., Wingfield B.D., De Vos L., van der Merwe N.A., Steenkamp N.A. (2021). Genome-wide Analysis of Repeat-Induced Point Mutations in the Ascomycota. Front. Microbiol..

[64] Amselem J., Quesneville H., Oliver R.P., Wincker P., Balesdent M.H., Howlett B.J. (2011). Effector Diversification within Compartments of the Leptosphaeria Maculans Genome Affected by Repeat-Induced Point mutations. Nat. Commun..

[65] Komluski J., Habig M., Stukenbrock E.H. (2023). Repeat-Induced Point Mutation and Gene Conversion Coinciding with Heterochromatin Shape the Genome of a Plant-Pathogenic Fungus. mBio.

[66] Van Wyk S., Harrison C.H., Wingfield B.D., De Vos L., van der Merwe N.A., Steenkamp E.T. (2019). The RIPper, a Web-based Tool for Genome-wide Quantification of Repeat-Induced Point (RIP) Mutations. PeerJ.

[67] Hartmann F.E., Sánchez-Vallet A., McDonald B.A., Croll D.A. (2017). Fungal Wheat Pathogen Evolved Host Specialization by Extensive Chromosomal Rearrangements. ISME J..

[68] Selker E.U. (1990). Premeiotic Instability of Repeated Sequences in *Neurospora crassa*. Annu. Rev. Genet..

[69] Selker E.U. (2002). Repeat-induced Gene Silencing in Fungi. Adv. Genet..

[70] Selker E.U., Cambareri E.B., Jensen B.C., Haack K. (1987). Rearrangement of Duplicated DNA in Specialized Cells of Neurospora. Cell.

[71] Galagan J.E., Selker E.U. (2004). RIP: The Evolutionary Cost of Genome Defense. Trends Genet..

[72] Son H., Min K., Lee J., Raju N.B., Lee Y.W. (2011). Meiotic Silencing in the Homothallic Fungus *Gibberella zeae*. Fungal Biol..

[73] Sridhar P.S., Trofimova D., Subramaniam R., González-Peña Fundora D., Foroud N.A., Allingham J.S., Loewen M.C. (2020). Ste2 receptor-mediated chemotropism of *Fusarium graminearum* Contributes to its Pathogenicity against Wheat. Sci. Rep..

[74] Urban M., King R., Hassani-Pak K., Hammond-Kosack K.E. (2015). Whole-genome Analysis of *Fusarium graminearum* Insertional Mutants Identifies Virulence Associated Genes and Unmasks Untagged Chromosomal Deletions. BMC Genom..

[75] King R., Urban M., Hammond-Kosack M.C., Hassani-Pak K., Hammond-Kosack K.E. (2015). The completed genome sequence of the pathogenic ascomycete fungus *Fusarium graminearum*. BMC Genom..

[76] Kim H.K., Jo S.M., Kim G.Y., Kim D.W., Kim Y.K., Yun S.H. (2015). A Large-Scale Functional Analysis of Putative Target Genes of Mating-Type Loci Provides Insight into the Regulation of Sexual Development of the Cereal Pathogen *Fusarium graminearum*. PLoS Genet..

[77] Irelan J.T., Selker E.U. (1997). Cytosine Methylation Associated with Repeat-induced Point Mutation Causes Epigenetic Gene Silencing in *Neurospora crassa*. Genetics.

[78] Hane J.K., Oliver R.P. (2010). In silico Reversal of Repeat-induced Point Mutation (RIP) Identifies the Origins of Repeat Families and Uncovers Obscured Duplicated Genes. BMC Genom..

[79] Chen L., Wang H., Yang J., Yang X., Zhang M., Zhao Z., Fan Y., Wang C., Wang J. (2021). Bioinformatics and Transcriptome Analysis of CFEM Proteins in *Fusarium graminearum*. J. Fungi.

[80] Cai N., Liu R., Yan D., Zhang N., Zhu K., Zhang D., Nong X., Tu X., Zhang Z., Wang G. (2022). Bioinformatics Analysis and Functional Characterization of the CFEM Proteins of *Metarhizium anisopliae*. J. Fungi.

[81] Xu M., Wang Q., Wang G., Zhang X., Liu H., Jiang C. (2022). Combatting *Fusarium head blight*: Advances in Molecular Interactions between *Fusarium graminearum* and Wheat. Phytopathol. Res..

[82] Rocher F., Alouane T., Philippe G., Martin M.L., Label P., Langin T., Bonhomme L. (2022). *Fusarium graminearum* Infection Strategy in Wheat Involves a Highly Conserved Genetic Program That Controls the Expression of a Core Effectome. Int. J. Mol. Sci..

[83] Hammond-Kosack K.E., Rudd J.J. (2008). Plant Resistance Signalling Hijacked by a Necrotrophic Fungal Pathogen. Plant Signal. Behav..

[84] Perincherry L., Lalak-Kańczugowska J., Stępień Ł. (2019). *Fusarium*-Produced Mycotoxins in Plant-Pathogen Interactions. Toxins.

[85] Zhao X.M., Zhang X.W., Tang W.H., Chen L. (2009). FPPI: *Fusarium graminearum* Protein-protein Interaction Database. J. Proteome Res..

[86] Bahrini I., Ogawa T., Kobayashi F., Kawahigashi H., Handa H. (2011). Overexpression of the Pathogen-inducible Wheat *TaWRKY45* Gene Confers Disease Resistance to Multiple Fungi in Transgenic Wheat Plants. Breed Sci..

[87] Buerstmayr M., Buerstmayr H. (2022). The Effect of the *Rht1* Haplotype on *Fusarium* head blight Resistance in Relation to Type and Level of Background Resistance and in Combination with *Fhb1* and *Qfhs.ifa-5A*. Theor. Appl. Genet..

[88] Mesterhazy A. (2024). What Is *Fusarium Head Blight* (FHB) Resistance and What Are Its Food Safety Risks in Wheat? Problems and Solutions—A Review. Toxins.

[89] Berraies S., Cuthbert R., Knox R., Singh A., DePauw R., Ruan Y., Bokore F., Henriquez M.A., Kumar S., Burt A. (2023). High-density Genetic Mapping of *Fusarium* head blight Resistance and Agronomic Traits in Spring Wheat. Front. Plant Sci..

[90] Buerstmayr M., Steiner B., Buerstmayr H. (2020). Breeding for *Fusarium* head blight Resistance in Wheat—Progress and Challenges. Plant Breed..

[91] Wang H., Sun S., Ge W., Zhao L., Hou B., Wang K., Lyu Z., Chen L., Xu S., Guo J. (2020). Horizontal gene transfer of *Fhb7* from fungus underlies *Fusarium* head blight resistance in wheat. Science.

[92] Su Z., Bernardo A., Tian B., Chen H., Wang S., Ma H., Cai S., Liu D., Zhang D., Li T. (2019). A deletion mutation in TaHRC confers *Fhb1* resistance to *Fusarium* head blight in wheat. Nat Genet..

