# Supplementary material for: Update on the Basic Understanding of Fusarium graminearum Virulence Factors in Common Wheat Research
Source: Plants (Basel). 2024 Apr 22;13(8):1159. doi: 10.3390/plants13081159 (PMC11053692; doi:10.3390/plants13081159)
Supplement: Supplementary file 1 [file plants-13-01159-s001.zip › plants-2822076-supplementary.docx]

**Table S1.** Previously studied effector genes of *Fusarium* *graminearum*, including their sub-cellular location, gene function, and phenotype mutation.

| Gene/ Protein | Gene function | Sub-Cellular location | Mutant phenotype | Reference |
| --- | --- | --- | --- | --- |
| Fgrab51 | Rab -GTPases | cytoplasm | loss of pathogenicity | 1 |
| Fgrab52 | Rab GTPases | cytoplasm | loss of pathogenicity | 1 |
| Fgrab6 | Rab GTPases | Golgi | loss of pathogenicity | 1 |
| Fgrab7 | Rab GTPases | vacuolar membrane | loss of pathogenicity | 1 |
| Fgrab8 | Rab GTPases | Tip of vegetative  hyphae and conidia | loss of pathogenicity | 1 |
| FGSG_00007 | O-methylsterigmatocystin oxidoreductase and cytochrome P450 monooxygenase or negative regulator of DON synthesis |  | increased virulence (hypervirulence) | 2 |
| FGSG_10397 |  |  | increased virulence (hypervirulence) | 2 |
| XM_390318 | ATF/CREB transcription factor | nucleus | increased virulence (hypervirulence) | 3 |
| ESU13482 | encode the catalytic subunits of cyclic AMP (cAMP)-dependent protein kinase A (PKA) |  | loss of pathogenicity | 4 |
| ESU14577 | encode the catalytic subunits of cyclic AMP (cAMP)-dependent protein kinase A (PKA) |  | loss of pathogenicity | 4 |
| FgSte50 | fungal development and pathogenicity | Cell membrane | loss of pathogenicity | 5 |
| Fgk3 | glycogen synthase kinase | cytoplasm | loss of pathogenicity | 6 |
| cdc2A | Cell cycle progression |  | loss of pathogenicity | 7 |
| MAP1 | Mitogen-activated protein kinase |  | loss of pathogenicity | 8 |
| FgGT2 | Glycosyltransferase |  | loss of pathogenicity | 9 |
| FGSG_12857.3 | Adenosine triphosphate (ATP) citrate lyase |  | loss of pathogenicity | 10 |
| FGSG_06039.3 | Adenosine triphosphate (ATP) citrate lyase |  | loss of pathogenicity | 10 |
| FgVam7 | Regulatory role in cellular differentiation and virulence, sexual, asexual growth | Cytoplasm, membrane | loss of pathogenicity | 11 |
| Fg02025 (FgArb1) | ATP-binding cassette (ABC) transporter | Cytoplasm, membrane | loss of pathogenicity | 12 |
| FGSG_10503 | Uncharacterized protein | vacuole membrane | loss of pathogenicity | 13 |
| FGSG_05094 | Uncharacterized protein |  | loss of pathogenicity | 13 |
| FGSG_09589 | 3-Isopropylmalate dehydratase |  | loss of pathogenicity | 14 |
| FGSG_12952 | 2-Isopropylmalate synthase |  | loss of pathogenicity | 14 |
| Tri12 | Suggested to play a role in export of trichothecene mycotoxins produced by Fusarium spp. | plasma membrane | reduced virulence | 15 |
| FGSG_01174.3 | Peroxisomal targeting signal receptor | cytoplasm | reduced virulence | 16 |
| FGSG_05596.3 | Peroxisomal biogenesis factor 6 | cytoplasm | reduced virulence | 16 |
| FgERG3A | C-5 sterol desaturase |  | reduced virulence | 17 |
| FgERG3B | C-5 sterol desaturase |  | reduced virulence | 17 |
| FgERG5A | cytochrome P450 61 |  | reduced virulence | 17 |
| fac1 | adenylate cyclase |  | reduced virulence | 18 |
| FgATF1 | stress-related transcription factor gene | nucleus | reduced virulence | 19 |
| FgSKN7 | stress-related transcription factor gene | nucleus | reduced virulence | 19 |
| Fgrac1 | Rho GTPases |  | reduced virulence | 20 |
| FGSG_05447 | Rho GTPases |  | reduced virulence | 20 |
| FGSG_04068 | Rho GTPases |  | reduced virulence | 20 |
| FGSG_00304 | Rho GTPases |  | reduced virulence | 20 |
| FgSNF1 | sucrose non-fermenting 1 protein kinase complex genes |  | reduced virulence | 21 |
| FgGAL83 | sucrose non-fermenting 1 protein kinase complex genes |  | reduced virulence | 21 |
| FgSNF4 | sucrose non-fermenting 1 protein kinase complex genes |  | reduced virulence | 21 |
| UBL1 | encoding a UBR-Box/RING domain E3 ubiquitin ligase |  | reduced virulence | 22 |
| MYT3 | encodes a putative Myb-like transcription factor; a transcriptional regulator | nuclei | reduced virulence | 23 |
| Chs2 | Chitin synthases |  | reduced virulence | 24 |
| Fgrab2 | Rab GTPases | Endoplasmic reticulum | reduced virulence | 25 |
| Fgrab4 | Rab GTPases | Early endosome | reduced virulence | 25 |
| FgVps35 | Components of retromer | cytoplasm | reduced virulence | 26 |
| FgVps26 | Components of retromer |  | reduced virulence | 26 |
| FgVps29 | Components of retromer |  | reduced virulence | 26 |
| FgVps17 | Components of retromer |  | reduced virulence | 26 |
| FgVps5 | Components of retromer |  | reduced virulence | 26 |
| Famyo2 | Myosin proteins |  | reduced virulence | 27 |
| FGSG_07546.3 | Putative Transcription Factor | Nuclear | reduced virulence | 28 |
| FGSG_12164 | Toxin Synthesis. pathogenicity and Reproduction |  | reduced virulence | 29 |
| FGSG_08634 | Global nitrogen regulator | Nucleus | reduced virulence | 30 |
| Fg01341 | Functionally reductant and that the calcineurin-Crz1-independent pathway | nucleus | Sexual reproduction blocked | 31 |
| Fg01341 | Functionally reductant and that the calcineurin-Crz1-independent pathway | Nucleus | Sexual reproduction blocked | 31 |
| FGSG_01503 | Regulators of G protein |  | reduced virulence | 32 |
| FGSG_06228 | Regulators of G protein |  | reduced virulence | 32 |
| CPK1 | encode the catalytic subunits of cyclic AMP (cAMP)-dependent protein kinase A (PKA) |  | reduced virulence | 33 |
| FgSho1 |  | Cell membrane | reduced virulence | 34 |
| FgSln1 | fungal development and pathogenicity | cell  membrane | reduced virulence | 34 |
| FgSte11 | fungal development and pathogenicity |  | reduced virulence | 34 |
| FgSte7 | fungal development and pathogenicity | Cell membrane | reduced virulence | 34 |
| FgABC1 | ABC pleiotropic drug resistance transporter, late infection stages in different tissues types |  | reduced virulence | 35 |
| FgSWI6 | transcription cofactor |  | reduced virulence | 36 |
| FGSG_02509.3 | Glycosylphosphatidylinositol-Anchored Proteins |  | reduced virulence | 37 |
| spt3 | Transcription factor |  | reduced virulence | 38 |
| spt8 | Transcription factor |  | reduced virulence | 38 |
| Fgac1 | Adenyly l Cyclase |  | reduced virulence | 39 |
| Chs3b | Chitin synthases |  | reduced virulence | 40 |
| FgSsn3 | Meiotic mRNA stability protein kinase, female sterile and defective in hypopodium formation and infectious growth |  | reduced virulence | 41 |
| FgVps27 | ESCRT-0 component, plays critical roles in a variety of cellular and biological processes |  | reduced virulence | 42 |
| FgHSP90 | Ubiquitous chaperone, heat shock protein 90 | nuclear | reduced virulence | 43 |
| FgTom1 | Tomatinase, a new virulence factor |  | reduced virulence | 44 |
| FgCAP1 | Adenylate-binding protein |  | reduced virulence | 45 |
| Pg1 | Endo-polygalacturonase, cell wall degrading enzymes |  | reduced virulence | 46 |
| FGSG_17662 | Major regulator of xylanase production |  | reduced virulence | 46 |
| FGSG_11011 | Endo-polygalacturonase |  | reduced virulence | 46 |
| FGSG_10089 | GPI-anchored cell wall protein |  | reduced virulence | 47 |
| FGSG_08737 | Woronin body protein, a glucokinase |  | reduced virulence | 47 |
| NoxA | NADPH oxidase |  | reduced virulence | 47 |
| NoxB | NADPH oxidase |  | reduced virulence | 47 |
| FgABCC9 (FG05_07325) | ABC-C transporter |  | reduced virulence | 48 |
| FgSec2A | Guanine nucleotide exchange factor |  | reduced virulence | 49 |
| FgRab8 | Member of Rab GTPase family |  | reduced virulence | 49 |
| FgSec2A | Guanine nucleotide exchange factor |  | reduced virulence | 49 |
| FgSec2B (FGSG_13119) | Guanine nucleotide exchange factor |  | reduced virulence | 49 |
| FgPLD1 (FGSG_09917) | Phospholipase | Cytoplasm | reduced virulence | 50 |
| FGRRES_07792 | Class X G-protein coupled receptor |  | reduced virulence | 51 |
| FgTfmI (FGSG_01555) | b-ZIP transcription factor, fungicide resistance |  | reduced virulence | 52 |
| FgEch1 (FGSG_13111) | Enoyl-CoA hydratase |  | reduced virulence | 53 |
| FgRav2 | Hypothetical protein of 318 amino acids, containing a ROGDI like leucine zipper domain known to have a regulatory role |  | loss of pathogenicity | 54 |
| AMT1 | Arginine Methyltransferase |  | reduced virulence | 55 |

References

1. Zheng, H.; Zheng, W.; Wu, C.; Yang, J.; Xi, Y.; Xie, Q.; Wang, Z. Rab GTPases are essential for membrane trafficking-dependent growth and pathogenicity in *Fusarium graminearum*. *Environ. Microbiol.* **2015**, *17*, 4580-4599.
2. Gardiner, D.M.; Kazan, K.; Manners, J.M.; Novel genes of Fusarium graminearum that negatively regulate deoxynivalenol production and virulence. *Mol. Plant*. *Microbe Interact*. **2009**, *22*, 1588-600.
3. Van Nguyen, T.; Kröger, C.; Bönnighausen, J.; Schäfer, W.; Bormann, J. The ATF/CREB Transcription Factor *Atf1* Is Essential for Full Virulence, Deoxynivalenol Production, and Stress Tolerance in the Cereal Pathogen *Fusarium graminearum*. *Mole. Plant Microbe Interact.* **2013**,  *26*, 1378-1394.
4. Hu, S.; Zhou, X.; Gu, X.; Cao, S.; Wang, C.; Xu, J.R. The cAMP-PKA Pathway Regulates Growth, Sexual and Asexual Differentiation, and Pathogenesis in *Fusarium graminearum*. *Mole. Plant Microbe. Interact.* **2014**. *27*, 557-566.
5. Gu, Q.; Chen, Y.; Liu, Y.; Zhang, C.; Ma, Z. The transmembrane protein FgSho1 regulates fungal development and pathogenicity via the MAPK module Ste50-Ste11-Ste7 in *Fusarium graminearum*. *New Phytol*. **2014**, *206*, 315-328.
6. Qin, J.; Wang, G.; Jiang, C.; Xu, J.R.; Wang, C. Fgk3 glycogen synthase kinase is important for development, pathogenesis and stress responses in *Fusarium graminearum*. *Sci. Rep.* **2015**, *5*, 08504.
7. Liu, H.; Zhang, S.; Ma, J.; Dai, Y.; Li, C.; Lyu, X.; Wang, C.; Xu, J.R.Two *Cdc2* Kinase Genes with Distinct Functions in Vegetative and Infectious Hyphae in *Fusarium graminearum*. *PLoS Pathog.* **2015**, *11*, e1004913.
8. Urban, M.; Mott, E.; Farley, T.; Hammond-Kosack, K. The *Fusarium graminearum* *MAP*1 gene is essential for pathogenicity and development of perithecia. *Mole. Plant Pathol.* **2003**, *4*, 347-359.
9. King, R.; Urban, M.; Lauder, R.P.; Hawkins, N.; Evans M.; Plummer A.; et al. A conserved fungal glycosyltransferase facilitates pathogenesis of plants by enabling hyphal growth on solid surfaces. *PLoS Pathog.* **2017**, *13*, e1006672.
10. Lee, S.; Son, H.; Lee, J.; Min, K.; Choi, G. J.; Kim, J.C.; Lee, Y.W. Functional Analyses of Two Acetyl Coenzyme A Synthetases in the Ascomycete Gibberella zeae. *Eukaryotic Cell*, **2011**, *10*, 1043-1052.
11. Zhang, H.; Li, B.; Fang, Q.; Li, Y.; Zheng, X.; Zhang, Z. SNARE protein FgVam7 controls growth, asexual and sexual development, and plant infection in *Fusarium graminearum*. *Mole. Plant Pathol.* **2015**, *17*, 108-119.
12. Yin, Y.; Wang, Z.; Cheng, D.; Chen, X.; Chen, Y.; Ma, Z. The ATP-binding protein FgArb1 is essential for penetration, infectious and normal growth of *Fusarium graminearum*. *New Phytol.* **2018**, *219*, 1447-1466.
13. Xie, Q.; Chen, A.; Zhang, Y.; Zhang, C.; Hu, Y.; Luo, Z.; Wang, Z.; ESCRT-III accessory proteins regulate fungal development and plant infection in *Fusarium graminearum*. *Curr Genet*. **2019**, *65*, 1041-1055.
14. Puri, K. D.; Yan, C.; Leng, Y.; Zhong, S. RNA-Seq Revealed Differences in Transcriptomes between 3ADON and 15ADON Populations of *Fusarium graminearum* In Vitro and In Planta. *PLOS ONE*, **2016**. *11*, e0163803.
15. Menke, J.; Dong, Y.; Kistler, H. C. *Fusarium graminearum* *Tri12p* Influences Virulence to Wheat and Trichothecene Accumulation. *Mole. Plant Microbe Interact.* **2012**, *25*, 1408-1418.
16. Min, K.; Son, H.; Lee, J.; Choi, G. J.; Kim, J.-C.; Lee, Y.-W. Peroxisome Function Is Required for Virulence and Survival of *Fusarium graminearum*. *Mol. Plant. Microbe Interact.* **2012**, *25*, 1617-1627.
17. Yun, Y.; Yin, D.; Dawood, D. H.; Liu, X.; Chen, Y.; Ma, Z. Functional characterization of FgERG3 and FgERG5 associated with ergosterol biosynthesis, vegetative differentiation and virulence of *Fusarium graminearum*. *Fungal Genetics Biol.* **2014**, *68*, 60-70.
18. Hu, S.; Zhou, X.; Gu, X.; Cao, S.; Wang, C.; Xu, J.R. The cAMP-PKA Pathway Regulates Growth, Sexual and Asexual Differentiation, and Pathogenesis in *Fusarium graminearum.* *Mol. Plant. Microbe Interact.* **2014**, *27*, 557-566.
19. Jiang, C.; Zhang, S.; Zhang, Q.; Tao, Y.; Wang, C.; Xu, J.R. *FgSKN7* and *FgATF1* have overlapping functions in ascosporogenesis, pathogenesis and stress responses in *Fusarium graminearum*. *Environ. Microb.* **2014**, *17*, 1245-60.
20. Zhang, C.; Wang, Y.; Wang, J.; Zhai, Z.; Zhang, L.; Zheng, W.; Wang, Z. Functional characterization of Rho family small GTPases in *Fusarium graminearum*. *Fungal. Genetics Biol.* **2013**, *61*, 90-99.
21. Yu, J.; Son, H.; Park, A. R.; Lee, S.H.; Choi, G. J.; Kim, J.C.; Lee, Y.W. Functional characterization of sucrose non-fermenting 1 protein kinase complex genes in the Ascomycete *Fusarium graminearum*. *Curr. Genet.* **2014***, 60,* 35-47*.*
22. Ridenour, J.B.; Smith, J.E.; Hirsch, R.L.; Horevaj, P.; Kim, H.; Sharma, S.; Bluhm, B.H. UBL1 of *Fusarium* verticillioides links the N-end rule pathway to extracellular sensing and plant pathogenesis. *Environ Microbiol*. **2014**, *16*, 2004-2022.
23. Kim, Y.; Kim, H.; Son, H.; Choi, G. J.; Kim, J.C.; Lee, Y.W. MYT3, A Myb-Like Transcription Factor, Affects Fungal Development and Pathogenicity of *Fusarium graminearum*. *PLoS ONE* **2014**, *9,* e94359
24. Cheng, W.; Song, X.S.; Li, H.P.; Cao, L.H.; Sun, K.; Qiu, X.L.; Liao, Y.C. Host-induced gene silencing of an essential chitin synthase gene confers durable resistance to *Fusarium head blight* and seedling blight in wheat. *Plant Biotechnol J.* **2015***, 13,* 1335-1345*.*
25. Zheng, H.; Zheng, W.; Wu, C.; Yang, J.; Xi, Y.; Xie, Q.; Wang, Z. Rab GTPases are essential for membrane trafficking-dependent growth and pathogenicity in *Fusarium graminearum*. *Environ Microbiol*. **2015**,17, 4580-4599.
26. Zheng, W.; Zheng, H.; Zhao, X.; Zhang, Y.; Xie, Q.; Lin, X.; Wang, Z. Retrograde trafficking from the endosome to the trans-Golgi network mediated by the retromer is required for fungal development and pathogenicity in *Fusarium graminearum*. *New Phytol.* **2016***, 210,* 1327-1343.
27. Zheng, Z.; Liu, X.; Li, B.; Cai, Y.; Zhu, Y.; Zhou, M. Myosins FaMyo2B and Famyo2 Affect Asexual and Sexual Development, Reduces Pathogenicity, and FaMyo2B Acts Jointly with the Myosin Passenger Protein FaSmy1 to Affect Resistance to Phenamacril in *Fusarium asiaticum*. *PLOS ONE* **2016**, *11*, e0154058.
28. Lin, Y.; Son, H.; Min, K.; Lee, J.; Choi, G. J.; Kim, J.C.; Lee, Y.W. A Putative Transcription Factor MYT2 Regulates Perithecium Size in the Ascomycete Gibberella zeae. *PLoS ONE* **2012**, *7*, e37859.
29. Jonkers, W.; Dong, Y.; Broz, K.; Corby Kistler, H. The Wor1-like Protein Fgp1 Regulates Pathogenicity, Toxin Synthesis and Reproduction in the Phytopathogenic Fungus *Fusarium graminearum*.  *PLoS Pathog.* **2012**, *8,* e1002724.
30. Hou, R.; Jiang, C.; Zheng, Q.; Wang, C.; Xu, J.R. The transcription factor mediates the regulation of deoxynivalenol (DON) synthesis by ammonium and cyclic adenosine monophosphate (cAMP) signalling in *Fusarium graminearum*. *Mol Plant Pathol.* **2015**, *16*, 987-999*.*
31. Zhang, X.; Cao, S.; Li, W.; Sun, H.; Deng, Y.; Zhang, A.; Chen, H. Functional Characterization of Calcineurin-Responsive Transcription Factors Fg01341 and Fg01350 in *Fusarium graminearum*. *Front. Microbiol*. **2020**, *11*, 597998.
32. Park, A. R.; Cho, A.R.; Seo, J.A.; Min, K.; Son, H.; Lee, J.; Lee, Y.W. Functional analyses of regulators of G protein signaling in Gibberella zeae. *Fungal. Genetics Biol.* **2012**, *49*, 511-520.
33. Hu, S.; Zhou, X.; Gu, X.; Cao, S.; Wang, C.; Xu, J.R. The cAMP-PKA Pathway Regulates Growth, Sexual and Asexual Differentiation, and Pathogenesis in *Fusarium graminearum*. *Mol. Plant. Microbe Interact.* , **2014**,  *27*, 557-566.
34. Gu, Q.; Chen, Y.; Liu, Y.; Zhang, C.; Ma, Z. The transmembrane protein FgSho1 regulates fungal development and pathogenicity via the MAPK module Ste50-Ste11-Ste7 in *Fusarium graminearum*. *New Phytol.* **2015**, *206*, 315-328*.*
35. Gardiner, D. M.; Stephens, A. E.; Munn, A. L.; Manners, J. M. An ABC pleiotropic drug resistance transporter of Fusarium graminearum with a role in crown and root diseases of wheat. *FEMS Microbiol Lett*. **2013**, *348*, 36-45.
36. Liu, N.; Fan, F.; Qiu, D.; Jiang, L. The transcription cofactor FgSwi6 plays a role in growth and development, carbendazim sensitivity, cellulose utilization, lithium tolerance, deoxynivalenol production and virulence in the filamentous fungus *Fusarium graminearum*. *Fungal. Genet. Biol*. **2013**, *59*, 42-52.
37. Rittenour, W. R.; Harris, S. D. Glycosylphosphatidylinositol-Anchored Proteins in *Fusarium graminearum*: Inventory, Variability, and Virulence. *PLoS ONE*, **2013**, *8*, e81603.
38. Gao, T.; Zheng, Z.; Hou, Y.; Zhou, M. Transcription factors *spt3* and *spt8* are associated with conidiation, mycelium growth, and pathogenicity in *Fusarium graminearum*. *FEMS Microbiol Lett*. **2014**, *351*, 42-50.
39. Bormann, J.; Boenisch, M. J.; Brückner, E.; Firat, D.; Schäfer, W. The Adenylyl Cyclase Plays a Regulatory Role in the Morphogenetic Switch from Vegetative to Pathogenic Lifestyle of *Fusarium graminearum* on Wheat. *PLoS ONE*, **2014**, *9*, e91135.
40. Cheng, W.; Song, X.S.; Li, H.P.; Cao, L.H.; Sun, K.; Qiu, X.L.; Liao, Y.C. Host-induced gene silencing of an essential chitin synthase gene confers durable resistance to *Fusarium head blight* and seedling blight in wheat. *Plant Biotechnol. J.* **2015**, 13, 1335-1345.
41. Cao, S.; Zhang, S.; Hao, C.; Liu, H.; Xu, J.R.; Jin, Q. FgSsn3 kinase, a component of the mediator complex, is important for sexual reproduction and pathogenesis in *Fusarium graminearum*. *Sci. Rep.* **2016***, 6,* 22333*.*
42. Xie, Q.; Chen, A.; Zhang, Y.; Yuan, M.; Xie, W.; Zhang, C. Zhou, J. Component Interaction of ESCRT Complexes Is Essential for Endocytosis-Dependent Growth, Reproduction, DON Production and Full Virulence in *Fusarium graminearum*. *. Front Microbiol.* **2019**, *10,* 180*.*
43. Bui, DC.; Lee, Y.; Lim, J. et al. Heat shock protein is required for sexual and asexual development, virulence, and heat shock response in *Fusarium graminearum*. *Sci Rep*, **2016**, *6*, 28154.
44. Carere, J.; Benfield, A. H.; Ollivier, M.; Liu, C. J.; Kazan, K.; Gardiner, D. M. A tomatinase-like enzyme acts as a virulence factor in the wheat pathogen *Fusarium graminearum*. *Fungal Genet. Biol.* **2017**, *100*, 33-41.
45. Yin T.; Zhang Q.; Wang J.; Liu H.; Wang C.; Xu JR.; Jiang C. The cyclase-associated protein FgCap1 has both protein kinase A-dependent and -independent functions during deoxynivalenol production and plant infection in *Fusarium graminearum*. *Mol.* *Plant Pathol.* **2018**, *19*, 552-563.
46. Paccanaro, M. C.; Sella, L.; Castiglioni, C.; Giacomello, F.; Martínez-Rocha, A. L.; D’Ovidio, R.; Favaron, F. Synergistic Effect of Different Plant Cell Wall–Degrading Enzymes Is Important for Virulence of *Fusarium graminearum*. *Mol. Plant. Microbe Interact.* **2017**, *30*, 886-895.
47. Fernando, U.; Chatur, S.; Joshi, M.; Bonner, C. T.; Fan, T.; Hubbard, K.; Rampitsch, C. Redox signalling from NADPH oxidase targets metabolic enzymes and developmental proteins in *Fusarium gramienarum*, *Mol. Plant Pathol.* **2019**, *20*, 92-106.
48. Qi, P.F.; Zhang, Y.-Z.; Liu, C.H.; Zhu, J.; Chen, Q.; Guo, Z.R.; Zheng, Y.L. Fusarium graminearum ATP-Binding Cassette Transporter Gene FgABCC9 Is Required for Its Transportation of Salicylic Acid, Fungicide Resistance, Mycelial Growth and Pathogenicity towards Wheat. *Int. J. Mol. Sci*. **2018**, *19*, 2351.
49. Zheng, H.; Li, L.; Miao, P.; Wu, C.; Chen, X.; Yuan, M.; Zhou, J. FgSec2A, a guanine nucleotide exchange factor of FgRab8, is important for polarized growth, pathogenicity and DON production in *Fusarium graminearum*. *Environ. Microbiol.* **2018**, *20,* 3378-3392*.*
50. Ding, M.; Zhu, Q.; Liang, Y.; Li, J.; Fan, X.; Yu, X.; Yu, J. Differential roles of three FgPLD genes in regulating development and pathogenicity in Fusarium graminearum. *Fungal Genet. Biol.* **2017**, *109,* 46-52*.*
51. Dilks, T.; Halsey, K.; De Vos, R. P.; Hammond-Kosack, K. E.; Brown, N. A. Non-canonical fungal G-protein coupled receptors promote Fusarium head blight on wheat. *PLoS Pathog*. **2019**, *15*, e1007666.
52. Liu, N.; Wu, S.; Dawood, D. H.; Tang, G.; Zhang, C.; Liang, J.; Ma, Z. The b‐ZIP transcription factor FgTfmI is required for the fungicide phenamacril tolerance and pathogenecity in *Fusarium graminearum*.  *Pest Manag. Sci.* **2019**, *75,* 3312-3322.
53. Tang, L.; Yu, X.; Zhang, L.; Zhang, L.; Chen, L.; Zou, S.; Dong, H. Mitochondrial FgEch1 is responsible for conidiation and full virulence in *Fusarium graminearum*. *Curr. Genet.* 2020, *66,* 361-371*.*
54. Spanu, F.; Scherm, B.; Camboni, I.; Balmas, V.; Pani, G.; Oufensou, S.; Migheli, Q. *FcRav2*, a gene with a ROGDI domain involved in Fusarium head blight and crown rot on durum wheat caused by *Fusarium culmorum*. *Mol. Plant Pathol.* **2018**, *19,* 677-688*.*
55. Wang, G.; Wang, C.; Hou, R.; Zhou, X.; Li, G.; Zhang, S.; Xu, J.R. The AMT1 Arginine Methyltransferase Gene Is Important for Plant Infection and Normal Hyphal Growth in *Fusarium graminearum*. *PLoS One* **2012**, *7*, e38324.
